# Reliability, Validity and Responsiveness of the EQ-5D-5L in Assessing and Valuing Health Status in Adolescents and Young Adults with Posttraumatic Stress Disorder: a Randomized Controlled Trail

**DOI:** 10.1007/s11126-020-09814-6

**Published:** 2020-08-17

**Authors:** Judith Dams, Eline Rimane, Regina Steil, Babette Renneberg, Rita Rosner, Hans-Helmut König

**Affiliations:** 1 Department of Health Economics and Health Services Research, Hamburg Center for Health Economics (HCHE), University Medical Center Hamburg-Eppendorf, Martinistraße 52, 20246 Hamburg, Germany; 2 Department of Psychology, Catholic University Eichstätt-Ingolstadt, Eichstätt, Germany; 3 Department of Clinical Psychology and Psychotherapy, Institute of Psychology, Goethe University Frankfurt, Frankfurt, Germany; 4 Department of Clinical Psychology and Psychotherapy, Freie Universitaet Berlin, Berlin, Germany

**Keywords:** Reliability, Validity, Responsiveness, EQ5D, Posttraumatic stress disorder

## Abstract

To validate the EQ-5D-5L health-related quality of life (HrQoL) questionnaire in adolescents and young adults with posttraumatic stress disorder (PTSD) after sexual and/or physical abuse. We used data from a randomized controlled trial of 87 participants with PTSD aged 14–21 years. The discriminative ability was evaluated by comparing participants scores on the descriptive system of the EQ-5D-5L with general population scores. Furthermore, the discriminative ability between subgroups of participants with different disease severity levels was estimated. Correlations between the EQ-5D index and clinical parameters were used to measure construct validity. Test-retest reliability was measured by intraclass correlation coefficients (ICC) between baseline, posttreatment and 3-month follow-up scores of participants with stable symptoms. Finally, the responsiveness of the EQ-5D was calculated by mean differences, effect sizes and receiver operating characteristic (ROC) analyses for participants with improved symptoms. Participants reported significantly more problems on the dimensions “mobility”, “usual activities”, “pain/discomfort” and “anxiety/depression” than the general population. The EQ-5D-5L was able to discriminate between different disease severity levels. The EQ-5D index and clinical scales were significantly correlated with absolute values of correlation coefficients varying between 0.21 and 0.59. Furthermore, ICCs between 0.65 and 0.91 indicated good test-retest reliability for the EQ-5D index. The ICCs for the EQ-VAS between baseline and 3-month follow-up, and posttreatment and 3-month follow-up were statistically significant with 0.71 and 0.87, respectively, whereas the ICC between baseline and posttreatment was 0.08 and not statistically significant. Effect sizes to measure the responsiveness ranged between −0.008 and 0.40 for the EQ-5D index and − 0.32 and 0.40 for the EQ-VAS. Furthermore, the area under the curve in ROC analyses was between 0.40 and 0.64 for the EQ-5D index and 0.60 and 0.70 for the EQ-VAS. Discriminative ability, test-retest reliability and construct validity of the EQ-5D-5L were good, whereas the responsiveness was rather weak. Nevertheless, the EQ-5D-5L can be used to measure HrQoL in adolescents and young adults with PTSD. German Clinical Trials Register identifier: DRKS00004787; date of registration: 18th March 2013; https://www.drks.de

**Electronic supplementary material:**

The online version of this article (10.1007/s11126-020-09814-6) contains supplementary material, which is available to authorized users.

## Background

Various studies address the high prevalence of sexual and/or physical abuse in children and adolescents and the mental consequences [1]. Studies estimate that 30% to 40% of sexually and/or physically abused children and adolescents develop posttraumatic stress disorder (PTSD) symptoms afterwards [2]. Patients with PTSD suffer from intrusive reliving of the traumatic event, and avoid stimuli associated with the trauma. Furthermore, patients show symptoms of hyperarousal, like difficulty in concentrating, hyper-vigilance or exaggerated startle response [3]. Consequences of PTSD are long-term and restrict the social life of patients. Thus, literature reported a reduced health related quality of life (HrQoL) of patients with PTSD [4–7].

In general, two methodological approaches to measure HrQoL can be distinguished. Symptoms and immediate consequences of the disease are captured by disease specific instruments, whereas generic instruments evaluate universal domains of HrQoL. Thus, disease specific instruments evaluate HrQoL in more detail, whereas generic instruments are used to compare HrQoL across different diseases, which is important to estimate quality-adjusted life years (QALYs) in health economic evaluations.

A frequently used generic instrument is the EQ-5D-5L [8, 9]. The EQ-5D-5L consists of five items addressing the dimensions ‘mobility’, ‘self-care’, ‘usual activities’, ‘pain/discomfort’, and ‘anxiety/depression’. Patients are asked to rate their problems in each dimension on an ordinal scale with the five levels “no problems”, “slight problems”, “moderate problems”, “severe problems” or “extreme problems”.

The usefulness of the EQ-5D-5L in measuring HrQoL can be evaluated by assessing its psychometric properties, comprising of the discriminative ability, construct validity, test-retest reliability and responsiveness. The discriminative ability refers to the ability of the instrument to discriminate between health states. The construct validity verifies whether the instrument measures what it is supposed to measure. The test-retest reliability is the ability to reproduce a result consistently in time, and the responsiveness examines the instrument’s reaction to changes in HrQoL.

Although psychometric properties of the EQ-5D for several mental disorders like schizophrenic disorders, anxiety disorders and social phobia have been analysed [10], those have not been evaluated for patients with PTSD yet. Therefore, this study aims to evaluate psychometric properties of the EQ-5D-5L in adolescents and young adults with PTSD.

## Methods

### Study Design and Participants

We used data collected within a multicentre randomized controlled trial (RCT) with adolescents and young adults with PTSD aged 14–21 years [11]. A primary diagnosis of abuse–related PTSD (with a lowered diagnostic threshold of two instead of three avoidance symptoms) was required for inclusion. Furthermore, participants had to have a sufficient knowledge of German language as well as safe living conditions. Participants with current severe suicidality or severe and life-threatening suicidality or self-harming behaviour within the last 6 month, IQ ≤ 75, current substance dependence (abstinence <6 months), or a substance-induced disorder, any documented pervasive developmental disorder, lifetime psychotic or bipolar disorder (unclear cases were included) according to the DSM-IV-TR [3], as well as simultaneous psychotherapeutic treatment, were excluded. Recruitment took place at German university outpatient clinics. At baseline (T0), no or a stable psychopharmacological treatment (≥ 3 weeks) was required. After intake participants were randomized either to a Developmentally adapted Cognitive Processing Therapy arm (D-CPT) or wait-list condition with treatment advice (WL/TA). Participant were re-evaluated subsequently at the end of treatment (T1, mean: 173 days after study entry; SD 42) and three months after the end of treatment (T2; mean: 261 days after study entry; SD 49). A detailed description of study design and treatments analysed in the RCT can be found elsewhere [11, 12]. The trial has been approved by the Ethics Committee of Catholic University Eichstätt-Ingolstadt, Frei Universitaet Berlin, and Goethe University Frankfurt, and is conducted according to the ICH Guideline for Good Clinical Practice.

### Health-Related Quality of Life

The EQ-5D-5L questionnaire was used to assess HrQoL [8, 9]. With the EQ-5D descriptive system, participants were asked to rate their health problems in the five dimensions ‘mobility’, ‘self-care’, ‘usual activities’, ‘pain/discomfort’ and ‘anxiety/depression’ on an ordinal five level scale with “no problems (1)”, “slight problems (2)”, “moderate problems (3)” , “severe problems (4)” or “extreme problems (5)”. Answers were combined to health states with “11111” and “55555” representing the best and worst health state, respectively. Overall, 3125 (5^5^) possible EQ-5D-5L health states exist. The EQ-5D index was calculated for the health state of each PTSD patient, such that health states were transformed to a scale between −0.661 representing the worst possible HrQoL, 0 representing death and 1 representing the best possible HrQoL using preference-based value sets derived from the German general population [13]. In addition to the descriptive system and EQ-5D index, HrQoL was assessed on the visual analogue scale of the EQ-5D (EQ-VAS). Participant were asked to rate their HrQoL visually between 0 (worst) and 100 (best HrQoL) [8]. For the current analyses, only participants with information on the EQ-5D-5L were included (*n* = 87).

### Socio-Demographics and Clinical Parameters

Socio-demographic variables included individual characteristics (e.g. age, gender) as well as family background, living situation and education. PTSD severity was measured by the clinical interview Clinician Administered PTSD Scale for Children and Adolescents (CAPS-CA) [14, 15], which rates frequency and intensity of PTSD symptoms on an ordinal scale ranging between 0 (never/none) to 4 (daily or almost daily/extreme with a total score ranging from 0 to 136). Additionally, self-reported PTSD symptoms were measured by the University of California Los Angeles PTSD Reaction Index (UCLA) with its total score ranging from 0 to 68 [16, 17]. Additionally, participants completed the Youth Self Report (YSR) with its total score ranging from 0 to 202 [18, 19]. The occurrence of borderline symptoms was measured by the Borderline Symptoms List-23 (BSL-23) (total score between 0 and 92) [20] and depressive symptoms were evaluated by the Beck Depression Inventory II (BDI-II) (total score between 0 and 63) [21, 22].

### Statistical Analysis

Statistical analysis comprised the (1) discriminative ability, (2) construct validity, (3) test-retest reliability, and (4) responsiveness of the EQ-5D-5L. Baseline data was used to assess the discriminative ability and the construct validity. Baseline, T1 and T2 data was used to test the test-retest reliability and the responsiveness of the EQ-5D-5L.

As the discriminative ability represents the ability to discriminate between different health states [23], the EQ-5D-5L scores of participants at baseline were compared with scores of the general population. These were taken from a telephone survey of 5005 adults representative for the German general population (≥ 18 years old) [24]. As participants with PTSD were aged 14–21 years, we only included general population respondents the age ≤ 21 (*n* = 257). In addition, the ability of the EQ-5D-5L to distinguish between different severity levels of PTSD was tested by subgroups depending on the CAPS-CA total score (*n* = 83). Subgroups were built based on tertiles of the CAPS-CA total scores with CAPS-CA total scores between 17 and 56 (*n* = 29), 57–73 (*n* = 27) and 74–113 (n = 27), respectively. Differences in the descriptive system scores of the EQ-5D-5L based on different severities of disease were tested by χ^2^-tests (α ≤ 0.05). Differences in the EQ-5D index and the EQ-VAS were tested using Mann-Whitney-U-tests (α ≤ 0.05).

The construct validity, i.e. the ability of the EQ-5D-5L to describe the underlying construct, which was assumed to be represented by PTSD and comorbid symptoms, was tested [25]. Baseline data was used to calculate correlations between symptoms scales (CAPS-CA, UCLA-PTSD-RI, YSR, BSL and BDI-II total score) and the EQ-5D index and EQ-VAS. As neither the EQ-5D index nor the EQ-VAS was distributed normally we used non-parametric Spearman rank correlation coefficients (r_s_). Small, moderate and large conformity between the EQ-5D index or the EQ-VAS and the CAPS-CA, UCLA, YSR, BSL and BDI-II total score were defined as 0.1 < |r_s_| ≤ 0.3, 0.3 < |r_s_| ≤ 0.5 and |r_s_| > 0.5, respectively [26].

The test-retest reliability was tested to investigate changes in the EQ-5D-5L between the different measurement points anchored by “no changes” in PTSD symptoms [25, 27]. Following previous literature [26, 28], “no changes” in the CAPS-CA total score between different measurement points were defined as CAPS-CA total score differences smaller than 0.5 standard deviations of the CAPS-CA score at baseline (±10.85 points on the CAPS-CA score). Intraclass correlation coefficients (ICCs) for EQ-5D index and EQ-VAS values were calculated with “no changes” between different measurement points. Similarity was assumed for an ICC ≥0.7 [25].

Responsiveness determines the ability of the EQ-5D-5L to detect changes in health states over time [29]. Thereby, changes between the different measurement points were anchored by “improvements” in PTSD symptoms, which were defined as CAPS-CA total score differences of more than 0.5 standard deviations of the CAPS-CA score at baseline. Mean differences of the EQ-5D index and EQ-VAS between the different measurement points were calculated and significance of “improvements” was tested by t-tests (α ≤ 0.05). Mean differences were standardized by the standard deviation at baseline to derive effect sizes (ES) or the standard deviation at the particular measurement point, T1 or T2, to derive standardized response means (SRM). According to Cohen, 0.1 ≤ |ES| or |SRM| < 0.2 represented almost no changes, 0.2 ≤ |ES| or |SRM| < 0.5 represented small changes, 0.5 ≤ |ES| or |SRM| < 0.8 represented medium changes and |ES| or |SRM| ≥ 0.8 represented large changes [26]. Finally, a receiver operating characteristic (ROC) analysis was conducted [30]. ROC analysis specifies whether participants with “improvements” in the CAPS-CA total score also improve in the EQ-5D index and the EQ-VAS. An instrument with perfect discrimination has an area under the curve (AUC) of 1.0, whereas random detection of changes will have an AUC of 0.5.

## Results

### Sample Characteristics

Sample characteristics are shown in Table 1. At baseline participants had a mean age of 18.1 (SD 2.3) years. Most of the participants were female (85%, *n* = 74) and were living at home with their parents (56%, *n* = 49). Smaller proportions of participants were living alone (15%, *n* = 13) or with other adolescents or young adults (18%, *n* = 16). Only 13% (*n* = 11) had achieved an A-level exam, because most of the participants had not quitted school yet due to their young age.

**Table 1:** Sample characteristics (*n* = 87)

Demographics			
age	mean | SD	18.1	2.3
male gender	n | %	13	14.9
A-level exam or higher	n | %	11	12.6
number of siblings	mean | SD	2.3	2.0
Living situation			
alone	mean | SD	13	14.9
with parents	mean | SD	49	56.3
emergency accommodation	on mean | SD	2	2.3
foster family	mean | SD	4	4.6
shared living	mean | SD	16	18.4
Symptom Severity			
UCLA total score	mean | SD	42.1	12.2
CAPS-CA total score	mean | SD	64.7	21.7
YSRs total score	mean | SD	71.7	21.9
BSL	mean | SD	37.1	18.6
BDI-II	mean | SD	29.1	13.8
Health related quality of life			
EQ-5D index	mean | SD	0.70	0.25
EQ-VAS	mean | SD	61.0	21.8

SD: standard derivation; n: number; %: percent

UCLA: University of California Los Angeles PTSD Reaction Index; CAPS-CA: Clinical Administered PTSD Scale for Children and Adolescent; YSR: Youth Self Report; BSL: Borderline Symptoms List-23; BDI-II: Beck Depression Inventory II;

### Clinical Characteristics

The mean severity of PTSD measured by the CAPS-CA and UCLA total score was 64.7 (SD 21.7) and 42.1 (SD 12.2), respectively. The mean YSR total score was 71.7 (SD 21.9). Furthermore, the mean BDI-II score was 29.1 (SD 13.8) and mean BSL score was 37.1 (SD 18.6).

The mean EQ-5D index was 0.7 (SD 0.3). Furthermore, participants reported a mean EQ-VAS of 61.0 (SD 21.8). Further details on socio-demographics and clinical characteristics are shown in Table 1.

### Discriminative Ability

Participants reported statistically significant more problems compared with persons of the general population on all dimensions of the EQ-5D descriptive system except self-care (Supplementary material: Fig. S1). 90% of the participants with PTSD reported problems due to anxiety/depression compared with only 24% of the persons of the general population. Problems due to pain/discomfort were reported by 74% of the participants with PTSD. Even though persons of the general population reported problems due to pain/discomfort less often (46%), this dimension of the EQ-5D descriptive system was impaired most frequently in the general population. Likewise, participants with PTSD had more problems in the dimensions usual activities and mobility (57% and 29% compared with 17% and 10%, respectively).

The ability of the EQ-5D-5L to distinguish between different severity levels of PTSD was tested. Participants with higher CAPS-CA total scores had lower EQ-5D index and EQ-VAS scores (Table 2). All correlations between the EQ-5D index and the CAPS-CA total score were statistically significant, whereas the EQ-VAS and CAPS-CA were only significantly correlated for CAPS-CA total scores between 57 and 73. Furthermore, the association between CAPS-CA total score and EQ-5D-5L descriptive system was not statistically significant.

**Table 2:** Discriminative ability: Descriptive statistics of the EQ-5D descriptive system, EQ-VAS and the EQ-5D index by disease severity at baseline

		Frequency of problems in EQ-5D dimensions (%)						
CAPS-CA total score	n	Morbidity	Self-care	Usual activities	Pain/discomfort	Anxiety/depression	EQ-5D index	EQ-VAS
17–56	29	6	1	11	17	21	0.85**	69.62
57–73	27	7	3	17	19	22	0.71***	59.93**
74–113	27	11	1	19	25	26	0.53*	52.88

SE: standard error; n: number; %: percent; Mann-Whitney-U-test *p*-value * ≤ 0.05

CAPS-CA: Clinical Administered PTSD Scale for Children and Adolescent

### Construct Validity

The EQ-5D index and the EQ-VAS were statistically significantly correlated with the CAPS-CA, as well as with the BDI-II and BSL scale (Table 3). Furthermore, the UCLA was statistically significantly correlated with the EQ-5D index, but not with the EQ-VAS. Thereby, |r_s_| ranged between 0.50 and 0.59 for the EQ-5D index and 0.21 and 0.40 for the EQ-VAS.

**Table 3:** Construct validity: Spearman rank correlation between EQ-5D index, EQ-VAS and scores of other instruments at baseline (n = 87)

Clinical instruments EQ-5D indexEQ-VAS		
CAPS-CA	−0.53***	−0.35**
UCLA	−0.50***	−0.21
YSR	−0.59***	−0.40**
BSL	0.54***	0.36**
BDI-II	−0.59***	−0.28**

n: number; Spearman rank correlation *p*-value * ≤ 0.05, ** ≤ 0.01, *** ≤ 0.001

CAPS-CA: Clinical Administered PTSD Scale for Children and Adolescent; YSR: Youth Self Report; BSL: Borderline Symptoms List-23; BDI-II: Beck Depression Inventory II;

### Test-Retest Reliability

ICCs were calculated to determine the test-retest reliability of the EQ-5D index and EQ-VAS anchored by “no changes” in the CAPS-CA total score (Table 4). Associations between different measurement points for the EQ-5D index were statistically significant with an ICC between 0.65 and 0.91. Associations between T2 and baseline or T1 for the EQ-VAS were statistically significant with an ICC between 0.71 and 0.87, respectively, whereas the association between baseline and T1 was not statistically significant.

**Table 4:** Test-retest reliability: Intraclass correlation coefficient (ICC) of the EQ-5D index and EQ-VAS anchored by “no changes” of the CAPS-CA total score (differences smaller than 0.5 standard deviations of the CAPS-CA score at baseline, which was equal to ±10.85 points on the CAPS-CA score)

		ICC	
Measurement points	n	EQ-5D index	EQ-VAS
Baseline – T1	10	0.73**	0.08
T1 – T2	30	0.91***	0.87***
Baseline – T2	8	0.65*	0.71*

n: number; Intraclass correlation coefficient *p*-value * ≤ 0.05, ** ≤ 0.01, *** ≤ 0.001

T1: posttreatment; T2: 3-month follow-up

CAPS-CA: Clinical Administered PTSD Scale for Children and Adolescent;

### Responsiveness

Analysis of responsiveness was anchored by “improvements” in the CAPS-CA total scores (≤ −10.85 points) and quantified by mean differences, ESs and SRMs of the EQ-5D index and EQ-VAS between the different measurement points (Table 5). Changes in the EQ-5D index between baseline and T1 were statistically significant with a mean difference of 0.09 (SD 0.11), an ES of 0.40 and a SRM of 0.78. Changes in the EQ-5D index between T1 and T2 were also statistically significant with mean difference of 0.04 (SD 0.09), an ES of 0.14 and a SRM of 0.44, whereas changes between baseline and T2 were not statistically significant. Furthermore, changes in the EQ-VAS were not statistically significant. ROC analyses revealed AUCs between 0.40 and 0.64 for the EQ-5D index and 0.60 and 0.70 for the EQ-VAS.

**Table 5:** Responsiveness: Improvement of health status measured by the EQ-5D index and EQ-VAS anchored by the CAPS-CA total score (<−10.25 points)

		Mean differences (SD)		Effect size (ES)		Standardized response mean (SRM)	
Measurement points	n	EQ-5D index	EQ-VAS	EQ-5D index	EQ-VAS	EQ-5D index	EQ-VAS
Baseline – T1	47	0.09 (0.11)*	8.9 (28.1)	0.40	0.40	0.78	0.32
T1 – T2	21	0.04 (0.09)*	0.8 (11.6)	0.14	0.04	0.44	0.07
Baseline – T2	43	−0.02 (0.19)	−7.0 (18.6)	−0.008	−0.32	−0.09	−0.38

n: number; SD: standard derivation; t-test p value * ≤ 0.05, ** ≤ 0.01, *** ≤ 0.001

T1: posttreatment; T2: 3-month follow-up

CAPS-CA: Clinical Administered PTSD Scale for Children and Adolescent;

## Discussion

### Discriminative Ability

As expected, participants with higher CAPS-CA total scores had lower EQ-5D index and EQ-VAS scores and reported more problems on the EQ-5D descriptive system, thus HrQoL was associated with the severity of PTSD. Compared with the general population participants with PTSD reported more problems in the dimensions anxiety/depression, pain/discomfort and usual activities. As participants with PTSD often suffer from pain/discomfort and anxiety/depression [31], likewise in adults [32], such impairments were expected for adolescents and young adults. Furthermore, literature reported impairments of HrQoL for adults with PTSD in daily life/social relationships, spare time activities and autonomy [33]. Thus, problems with usual activities were likely caused by direct consequences of PTSD. In contrast to mental comorbidities like anxiety/depression participants rarely suffer from somatic comorbidities due to their young age. Therefore, problems with self-care were expected to be low and similar to those of the general population.

The EQ-5D index was able to discriminate between different severities of PTSD, whereas the EQ-VAS was associated with the severity of PTSD only for participants with moderate CAPS-CA total scores. As this comparison strongly relies on the groups built, different group sizes were tested by using quartiles instead of tertiles as threshold for the CAPS-CA total score. However, results remained insignificant. Therefore, we expect the EQ-VAS to be less able to discriminate between different severities of PTSD than the EQ-5D index. As suggested for other mental disorders like schizophrenia or anxiety disorders before [10], it is more likely that the EQ-VAS addresses different concepts of HrQoL than clinical scales. However, differences in the discriminative ability between the EQ-5D index and EQ-VAS for participants with CAPS-CA total scores in the first and third tertile were difficult to explain.

### Construct Validity

The EQ-5D index was correlated with the CAPS-CA, UCLA, YSR, BDI-II total score, and the BSL total score, whereas the EQ-VAS was correlated with all clinical scales except the UCLA. According to Cohen, conformity was large between the EQ-5D index and clinical scales because all Spearman rank correlations |r_s_| were statistically significant and > 0.5 [26]. Conformity between the EQ-VAS and the CAPS-CA total score, YSR and BSL were moderate with 0.3 < |r_s_| ≤ 0.5 and small between the EQ-VAS and BDI-II total score with 0.3 ≤ |r_s_|. Interestingly, no conformity was found between the EQ-VAS and UCLA total score, even though the EQ-VAS was moderately correlated with the CAPS-CA total score. The CAPS-CA and UCLA are both able to measure symptoms of PTSD in children, adolescents and young adults based on the DSM-V criteria [34], thus differences in correlation with the EQ-VAS may rather be due to the different type of assessment. As the UCLA is a self-rated questionnaire, whereas the CAPS-CA is a clinician-rated interview, different perceptions of PTSD symptoms may influence correlations.

### Test-Retest Reliability

ICCs for the EQ-5D index and EQ-VAS for all pairwise comparisons of different measurement points anchored by “no changes” in the CAPS-CA were statistically significant, except for the comparison of the EQ-VAS between baseline and T1. Thereby, the EQ-VAS varied more in the CAPS-CA total score between baseline and T1 than the EQ-5D index. As the sample size of participants with “no changes” on the CAPS-CA total score between baseline and T1 was rather small (*n* = 10) and most of these participants were in the WL/TA condition (*n* = 8), results may be unreliable and further research is needed. Nevertheless, the test-retest reliability for both, the EQ-5D index and EQ-VAS, was good.

### Responsiveness

The EQ-5D index was able to represent improvements of the CAPS-CA total score between baseline and T1, which were clinically meaningful, as mean differences exceeded published threshold of minimal clinically important differences of the EQ-5D index for adults with PTSD of 0.05 to 0.08 [35]. However, for all other interactions between CAPS-CA improvements either changes in the EQ-5D index between measurement points were statistically insignificant or ES and SRM indicated no changes. Additionally, AUCs of ROC analyses ranged between 0.4 and 0.7, thus CAPS-CA improvements were hardly represented by the EQ-5D index or the EQ-VAS values. As treatment effects may be captured more precisely by symptom scales of PTSD than generic HrQoL measures, the CAPS-CA scale may be more sensitive to capture improvements compared with the EQ-5D. Overall, the responsiveness of the EQ-5D index and the EQ-VAS was weak, which might be caused by the treatment effects themselves and the small sample size of the study. In particular, as most WL/TA participants did not receive any treatment, changes between baseline, T1 and T2 were small, thus most of the participants did not improve in the CAPS-CA total score and were therefore not included in the responsiveness analysis.

### Strength and Limitations

Our study was based on the EQ-5D-5L, for which value sets were published recently to calculate the EQ-5D index [13]. Previously the EQ-5D-3L was available to determine HrQoL by rating problems on an ordinal scale with three levels. This version was extended to an ordinal scale with five levels, which therefore enables to evaluate HrQoL more precisely. Thus, this version is expected to replace the commonly used version of the EQ-5D-3L and will be of high relevance for future research. Furthermore, literature on HrQoL for adolescents and young adults with PTSD was lacking. Only one study reported data on HrQoL for children with PTSD aged between 9 and 11 years measuring the Life Satisfaction Scale [36].

However, our study has several limitations. First, the sample size of *n* = 87 was small, thus results might be preliminary, calling for further research. Second, participants with PTSD and persons of the general population differed in age. Participants with PTSD were aged between 14 and 21 years, whereas persons of the general population were between 18 and 21 years old. As we expect that HrQoL for younger persons is better or equal compared to older persons, younger persons of the general population would increase the discriminative ability of the EQ-5D-5L. Finally, mean differences, ESs and SRMs indicated a low responsiveness for the EQ-5D index and EQ-VAS, thus the EQ-5D might not represent small changes in clinical symptoms over time.

## Conclusion

The EQ-5D-5L is a valid instrument to measure HrQoL in adolescents and young adults with PTSD. The discriminative ability, the construct validity, and the test-retest reliability were good, whereas the responsiveness was rather weak.

## Electronic supplementary material

ESM 1 (DOCX 32 kb)

## Data Availability

Data from patients cannot be accessed by anyone who is not part of the research team due to ethical and confidentiality concerns.

## Abbreviations

AUC: Area under the curve
BDI-II: Beck Depression Inventory II
BSL-23: Borderline Symptoms List-23
CAPS-CA: Clinician Administered PTSD Scale for Children and Adolescents
D-CPT: Developmentally Adapted Cognitive Processing Therapy
EQ-5D: EuroQoL-5 Dimension
EQ-VAS: Visual Analogue Scale of the EQ-5D
ES: Effect sizes
HrQoL: Health related quality of life
ICC: Intraclass correlation coefficient
PTSD: Posttraumatic stress disorder
ROC: Receiver operating characteristic
SRM: Standardized response means
UCLA: University of California Los Angeles
PTSD: Reaction Index
WL/TA: Wait-list condition with treatment advice
YSR: Youth Self Report
