# Supplementary material for: Reliability, Validity and Responsiveness of the EQ-5D-5L in Assessing and Valuing Health Status in Adolescents and Young Adults with Posttraumatic Stress Disorder: a Randomized Controlled Trail
Source: Psychiatr Q. 2020 Aug 17;92(2):459–71. doi: 10.1007/s11126-020-09814-6 (PMC8110499; doi:10.1007/s11126-020-09814-6)
Supplement: Supplementary file 1 — (DOCX 32 kb) [file 11126_2020_9814_MOESM1_ESM.docx]

**Supplementary material:**

**Figure S1: Discriminative ability**

Comparison of the EQ-5D dimensions between participants sample (n=87) and general population (n=257)
